# Effects of *Satureja Khuzestanica* supplementation on glycemic indices and lipid profile in type 2 diabetes patients: a randomized controlled clinical-trial

**DOI:** 10.1186/s12906-024-04384-7

**Published:** 2024-05-22

**Authors:** Sajjad Roosta, Fatemeh Ghasemi, Yaser Mokhayeri, Saeed Choobkar, Mohammad Reza Nikbakht, Ebrahim Falahi

**Affiliations:** 1 Student Research Committee, Lorestan University of Medical Sciences, Khorramabad, Iran; 2 Cardiovascular Research Center, Shahid Rahimi Hospital, Lorestan University of Medical Sciences, Khorramabad, Iran; 3 School of Medicine, Lorestan University of Medical Sciences, Khorramabad, Iran; 4 Department of Physiology and Pharmacology, School of Medicine, Yasuj University of Medical Sciences, Yasuj, Iran; 5 Nutritional Health Research Center, Lorestan University of Medical Sciences, P.O. Box: 6819789741, Khorramabad, Iran

**Keywords:** Diabetes mellitus, Fasting blood glucose, *Satureja Khuzestanica*

## Abstract

**Background:**

Several studies showed the hypoglycemic and hypolipidemic effects of *Satureja Khuzestanica* (SK) in animal models. This study aimed to determine the effect of SK supplementation on glycemic and lipid outcomes of patients with type 2 diabetes mellitus (T2DM).

**Methods:**

The study was designed as a double-blind, placebo-controlled, randomized clinical trial using block randomization. Seventy-eight T2DM patients were randomly assigned to intervention (*n* = 39) or placebo (*n* = 39) groups. They received SK or placebo in 500 mg capsules daily for 12 weeks. Anthropometric, blood pressure, liver enzymes, glycemic, and lipid outcomes were measured before and after the intervention.

**Results:**

At baseline, there were no significant differences in age, sex, or glycated hemoglobin (HbA1c) levels between the groups. SK supplementation led to a significant decrease in FBS (-12.6 ± 20.7 mg/dl in the intervention group versus 3.5 ± 31.9 mg/dl; *p* = 0.007), HbA1c (-0.28 ± 0.45 in the intervention group versus 0.11 ± 0.54% in the placebo group; p = < 0.001), insulin (-1.65 ± 6.18 in the intervention group versus 2.09 ± 5.90 mIU/L in the placebo group; *p* = 0.03), total cholesterol (-14.6 ± 21.1 mg/dl in the intervention group versus 8.2 ± 30.9 mg/dl in the placebo group; *p* < 0.001), LDL-cholesterol (-4.6 ± 15.2 mg/dl in the intervention group versus 5.8 ± 14.6 mg/dl in placebo group; *p* < 0.001) levels, and significant increase in HDL-cholesterol (3.9 ± 4.9 mg/dl in the intervention group versus 0.9 ± 5.2 mg/dl in placebo group; *p* = 0.005).

**Conclusion:**

Based on the study results, SK supplementation may improve glycemic indices and lipid profile of patients with T2DM. Our findings may provide novel complementary treatments without adverse effects for diabetes complications. These results need to be further confirmed in clinical trials.

**Registration:**

: This trial has been registered in the Iranian Registry of Clinical Trials (IRCT ID: IRCT20190715044214N1, registration date: 21/02/2021).

## Background

Diabetes is a multifactorial disease characterized by impaired metabolism of carbohydrates, lipids, and proteins and is caused by insulin resistance, impaired insulin secretion, or a combination of both [1]. In 2015, it was estimated that 1 in 11 adults globally had diabetes mellitus [2]. The diabetes burden has been increasing in recent years. The prevalence rate of type 2 diabetes was 6059 cases per 100,000 people in 2017, or about 462 million people [3]. The ninth leading cause of death is diabetes mellitus alone, which accounts for more than 1 million deaths per year [4]. At around 55 years of age, the incidence of diabetes mellitus peaks. It affected women and men equally [3]. By 2030, the diabetes prevalence worldwide is predicted to reach 10.2% (578 million people), an increase that will be seen across all world regions [5].

Despite the benefits of blood glucose-lowering medications, including oral agents and exogenous insulin, in controlling the progression of type 2 diabetes and its complications, the safety of these drugs has been recently debated due to their side effects [6].

Diverse complementary, alternative, and traditional treatment approaches have been increasingly used in developing and developed countries to treat chronic diseases such as diabetes [7]. Complementary and alternative medicine is a term for remedies and practices not deemed part of conventional medicine [8]. Herbal medicine is one of the most important of these approaches. People have been using medicinal herbs since ancient times. Some of them are used as complementary therapies [9]. Complementary and alternative treatments, mainly medicinal herbs, have been suggested to improve glycemic control and cardiovascular disease risk in diabetic patients [10, 11]. Among medicinal plants used for diabetes, *Satureja Khuzestanica* (SK), an endemic annual plant distributed in southwestern Iran, has been used for its beneficial effects and has been somewhat investigated. It also has been traditionally used as a liver tonic herb [12].

Various studies have reported different beneficial effects of *SK* on health, including antimicrobial and antifungal activity, antinociceptive and anti-inflammatory properties, and vasodilation effects [13–16]. Several animal and human studies have investigated its anti-diabetic effects with contradictory results [15, 17–20]. Animal studies have demonstrated that *SK* lowers plasma glucose, total cholesterol, LDL-cholesterol, and triglyceride levels and increases HDL-cholesterol levels in diabetic rats [15, 19, 20]. To our knowledge, only one randomized controlled clinical trial has investigated the diabetic effects of *SK*; its results revealed a significant decrease in total cholesterol and LDL-cholesterol levels and an increase in HDL-cholesterol. Contrariwise, it exhibited no effects on plasma glucose and triglyceride levels [21]. However, this study had some limitations, including a small sample size, short duration of supplementation, and not adjusting for baseline variables.

Despite the positive results on anti-diabetic activities of *SK* in animal models, these activities have not yet been thoroughly examined in humans. So, this study aimed to determine the effects of *SK* supplementation on glycemic control indices, lipid profile, and liver enzymes in patients with type 2 diabetes mellitus.

## Methods

### Study design and subjects

In this double-blinded, randomized controlled clinical trial study, seventy-eight patients with type 2 diabetes mellitus (T2DM) aged between 18 and 60 years were recruited for this study from an endocrinology clinic in Khorramabad, Iran. For at least a year, all of the patients had been diagnosed with type 2 diabetes mellitus. Other eligibility criteria were a body mass index (BMI) between 18.5 and 40 kg/m^2^, lack of comorbid uncontrolled diseases, taking oral hypoglycemic medications for diabetes, not being on a weight loss or weight gain diet, and lack of pregnancy and lactation for women. Patients were excluded if they exhibited an adverse reaction to *SK*, altered their treatment during the study, and required insulin injections.

**Fig. 1:**
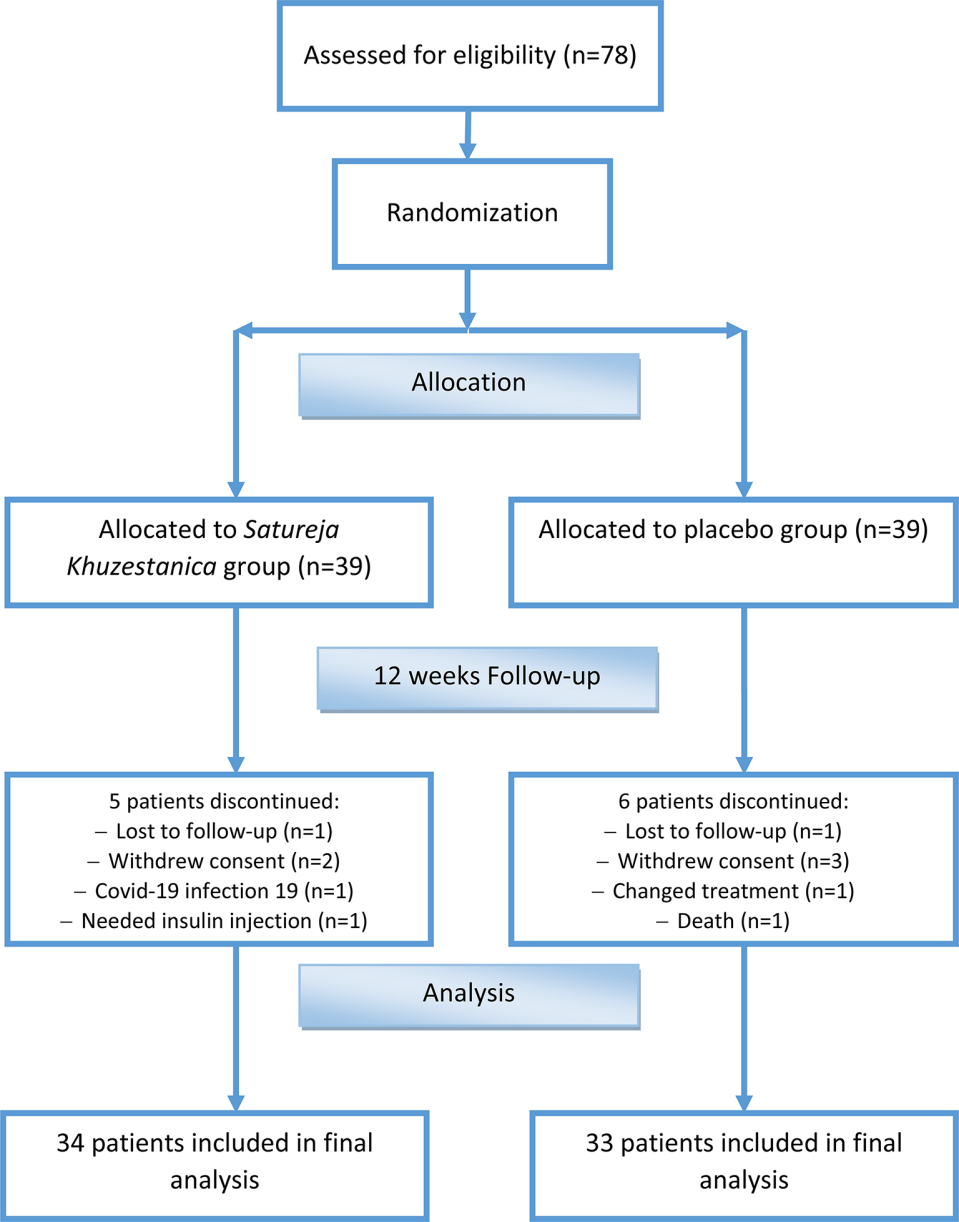
Study flow

The flow of allocation, follow-up, and analysis of the study is shown in Fig. 1.

### Sample size

The sample size in each study arm was estimated to be 32 people using the following formula [22], considering the alpha error of 0.05 and beta error of 0.20, and based on the mean changes of HDL-c in the intervention and control groups of the previous study [21]. Considering the dropout rate of 20%, 39 people enrolled in each study arm; therefore, the total sample size was 78.

$$N\,=\,2\, \times \,\left( {\frac{{{z_{1\, - \,\alpha }}\,+\,{z_{1\, - \,\beta }}}}{{\delta \, - \,{\delta _0}}}} \right)\, \times \,{s^2}$$

### Randomization and blinding

This study randomly allocated individuals to the intervention or control groups using a block randomization approach. Based on their group, patients who registered in the trial got three bottles of capsules. Each bottle contained 28 capsules. Both *SK* and placebo capsules were packaged in similar bottles. Someone uninvolved in patients’ visits, allocation, or follow-up filled the capsules into the bottles.

A follow-up phone call was made biweekly to emphasize taking the drugs and checking for any potential side effects. The participants were visited 12 weeks later and asked to return the remaining capsules. If more than 20% of them were left, the patient would be excluded from the study.

No information on the contents of the bottles was provided to the physician, the patient, and the person in charge of group allocation.

### Preparation of the supplement and placebo

The aerial parts of the SK were collected during the flowering stage. The preparation and drying of the plants were done by Khorraman Co. (Khorramabad, Iran). Dried leaves of SK were thoroughly ground in a mechanical grinder. The obtained powder was filled in 500 mg capsules. In parallel, placebo capsules were prepared containing the same amount of talcum powder, made of hydrated magnesium silicate with the chemical formula Mg_3_Si_4_O_10_(OH)_2_. Both SK and placebo capsules were packaged in similar bottles.

### Measurements

Patients were informed about the study’s topic, objectives, and methods. Trained interviewers collected information on age, sex, dietary intake (based on 24-hour food recall), medical history, medication use, and smoking habits. Anthropometric indices and clinical and biochemical outcomes were assessed using the following methods once patients were finalized based on eligibility criteria and signed the informed written consent at the baseline and 12 weeks after the intervention.

We assessed food intakes using 24-hour food recall for three days (including a weekend day). Patients were asked not to change their diet during the study.

The modified Nutritionist IV program calculated nutrient intakes from the 24-hour food recall.

Physical activity was assessed using the International Physical Activity Questionnaire (IPAQ). The short version of IPAQ (seven items) containing information on weekly time spent walking, vigorous, moderate, intense, and sedentary activity was used. Finally, metabolic equivalent minutes per week (MET -min/wk.) were calculated, and their sum was determined as physical activity.

Weight was measured using a digital scale (SECA, Germany) with a precision of up to 0.1 kg, while patients wore light clothes and no shoes or socks. Patients’ heights were measured using a stadiometer (SECA, Germany) with a 0.5 cm precision in a standing position with the neck straight and looking straight ahead and shoulders in normal alignment. BMI was calculated by dividing weight in kilograms by height in meters squared. Waist circumference was measured using an unstretched shape tape meter to the nearest 0.1 cm precision at the level of the umbilicus and without any pressure.

Blood pressure was measured twice using a mercury sphygmomanometer ALPK2 (Japan) and the Korotkoff sound technique, with an interval of 3 min, while the patient was sitting in a chair and rested for at least 15 min; the final pressure of the patient was calculated as the average of two measurements.

Blood samples were taken from all subjects after 12–14 h of overnight fasting between 7:00 and 9:00 a.m. All blood samples were centrifuged within 30 to 45 min of collection. Fasting blood sugar (FBS) was measured using the standard enzymatic method with Pars Azmun Co. assay kits (Tehran, Iran). The concentrations of serum triglyceride (TG), total cholesterol (TC), HDL-cholesterol, LDL-cholesterol, alanine aminotransferase (ALT), and aspartate aminotransferase (AST) were measured by colorimetric techniques using commercial kits (Pars-Azmun Co., Tehran, Iran). All the blood samples were analyzed by Selectra ProM auto-analyzer. The glycosylated hemoglobin (HbA1c) was measured by a commercial HbA1c kit (Pars Azmun, Tehran, Iran). The fasting insulin concentration was determined using a commercial enzyme-linked immunosorbent assay (ELISA) kit (Monobind, California, USA).

### Intervention

All patients received three bottles of *SK* capsules (containing 500 mg of dried leaves) or Placebo capsules (containing 500 mg of dried leaves) or Placebo capsules (500 mg of talcum powder). They were asked to take their capsules daily after lunchtime for 12 weeks. A biweekly follow-up phone call emphasized taking the capsules and monitoring for potential adverse effects. Participants were allowed to leave the study if they experienced any side effects or were dissatisfied with continuing the study. After 12 weeks, subjects’ anthropometric indices and clinical and biochemical outcomes were measured again.

The flow of allocation, follow-up, and analysis of the study is shown in Fig. 1.

### Statistical analyses

All analyses were performed using Stata version 16. Data are represented as the mean ± SD. An independent t-test was used to compare the mean of variables in the study groups before the intervention. The analysis of covariance (ANCOVA) test was used to compare the mean of outcome variables between the groups, with adjustment of baseline values as possible confounding variables. P values less than 0.05 were considered statistically significant.

## Results

Seventy-eight eligible patients were randomly assigned to the *SK* and placebo groups. Five patients in the *SK* group and six in the placebo group discontinued the study for different reasons. An analysis of patient data was conducted at the end of the study, which involved 34 patients in the *SK* group and 33 patients in the placebo group.

Table 1 shows patients’ baseline demographic, anthropometric, biochemical, and clinical characteristics, as well as macronutrient intake, in the two groups. There were no statistically significant differences between the two groups of patients.

**Table 1:** Baseline characteristics of patients with type 2 diabetes mellitus in *Satureja Khuzestanica* and placebo groups

Characteristics	Satureja Khuzestanica Mean ± SD (*n* = 34)	Placebo Mean ± SD (*n* = 33)	p-value^*^
Gender (female/male)	21/13	21/12	0. 87
Age (year)	55.5 ± 7.4	54.7 ± 6.4	0.75
Diabetes duration (year)	6.1 ± 3.9	8 ± 5.4	0.11
Weight (Kg)	78.7 ± 13.2	80.1 ± 13.7	0.69
BMI (Kg/m^2^)	29.4 ± 4.4	29.6 ± 4.2	0.94
WC (cm)	102.9 ± 8.5	101.7 ± 7.1	0.91
FBS (mg/dl)	163.9 ± 28.4	165 ± 23.3	0.86
HbA_1_c (%)	7.7 ± 0.8	7.9 ± 0.7	0.27
Insulin (mIU/L)	16.5 ± 16.9	12.5 ± 6.7	0.20
TG (mg/dl)	213.1 ± 86.5	219 ± 109.5	0.79
Total Cholesterol (mg/dl)	215.1 ± 41.7	209.2 ± 49.8	0.87
LDL-C (mg/dl)	120.2 ± 28.7	123 ± 28.1	0.92
HDL-C (mg/dl)	40.7 ± 9.4	38.3 ± 7.4	0.26
ALT (mg/dl)	23.08 ± 10.66	28.1 ± 14.9	0.11
AST (mg/dl)	25.1 ± 10.8	26.4 ± 11.3	0.66
SBP (mmHg)	127.3 ± 13.7	124.3 ± 11.1	0.33
DBP (mmHg)	80.7 ± 5.9	81.7 ± 6	0.84
Total energy intake (kcal/day)	2128.1 ± 565.2	2213 ± 447.3	0.49
Dietary carbohydrate intake (g/day)	249.8 ± 77.6	271.1 ± 70.8	0.24
Dietary protein intake (g/day)	79.2 ± 28.4	79.3 ± 26	0.98
Dietary fat intake (g/day)	94.3 ± 38.4	95.1 ± 23.1	0.86
Physical activity (MET min/week)	605.1 ± 705.7	581.3 ± 452.3	0.87

BMI, Body mass index; WC, Waist circumferences; FBS, Fasting blood sugar; TG, Triglyceride; LDL-C, Lo-density lipoprotein cholesterol; HDL-C, High-density lipoprotein cholesterol; ALT, Alanine Aminotransferase; AST, Aspartate Aminotransferase; SBP, Systolic blood pressure; DBP, Diastolic blood pressure

^*^Independent t-test was used

Changes in the outcomes of the two groups are shown in Table 2. A significant decrease in weight, fasting blood sugar, insulin, total cholesterol, and LDL cholesterol levels and a significant increase in HDL cholesterol levels were observed in patients in the SK group.

**Table 2:** Changes in outcomes of patients with type 2 diabetes mellitus in *Satureja Khuzestanica* and placebo groups

Characteristics	Satureja Khuzestanica Mean ± SD (*n* = 34)	Placebo Mean ± SD (*n* = 33)	p-value^*^
Weight (Kg)	-0.72 ± 1.57	-0.14 ± 1.23	0.03
BMI (Kg/m^2^)	-0.25 ± 0.59	-0.04 ± 0.44	0.07
WC (cm)	-1.15 ± 2.23	-0.35 ± 1.65	0.06
FBS (mg/dl)	-12.62 ± 20.78	3.48 ± 31.95	0.007
HbA_1_c (%)	-0.28 ± 0.45	0.11 ± 0.54	< 0.001
Insulin (mIU/L)	-1.65 ± 6.18	2.09 ± 5.90	0.03
TG (mg/dl)	-13.26 ± 33.78	-6.06 ± 35.93	0.14
Total Cholesterol (mg/dl)	-14.56 ± 21.12	8.15 ± 30.96	< 0.001
LDL-C (mg/dl)	-4.62 ± 15.27	5.76 ± 14.45	< 0.001
HDL-C (mg/dl)	3.94 ± 4.99	0.94 ± 5.26	0.005
ALT (mg/dl)	-0.53 ± 6.75	-0.91 ± 6.20	0.20
AST (mg/dl)	0.97 ± 6.29	2 ± 6.05	0.29
SBP (mmHg)	-2.18 ± 6.05	-0.85 ± 4.63	0.57
DBP (mmHg)	-1.21 ± 3.01	-0.51 ± 3.42	0.22

BMI, Body mass index; WC, Waist circumferences; FBS, Fasting blood sugar; TG, Triglyceride; LDL-C, Lo-density lipoprotein cholesterol; HDL-C, High-density lipoprotein cholesterol; ALT, Alanine Aminotransferase; AST, Aspartate Aminotransferase; SBP, Systolic blood pressure; DBP, Diastolic blood pressure

^*^ANCOVA test was used

## Discussion

In this study, supplementation of patients with diabetes with *SK* (daily dose of 500 mg) improved weight, glycemic indices (FBS, HbA1C, and Fasting Insulin), and lipids (total cholesterol, LDL-c, and HDL-c) outcomes (except for triglycerides level) in the intervention group compared to the placebo group.

The improvements observed in this study on glycemic indices and lipid profile of patients with diabetes were consistent with those observed in previous studies (in decreasing FBS [18, 20, 23], insulin [19], increasing HDL and reduction of LDL and total cholesterol [18, 20]. Conversely, a clinical trial study has failed to demonstrate such improvements in glycemic control; however, in terms of improving lipid profile, it showed results similar to the present study [21]. A possible reason for the inconsistency in glycemic control between their study and the current study could be the different doses and duration of interventions.

Different mechanisms may be involved in the observed hypoglycemic effects of *SK*. It has been reported that *SK* essential oil decreases phosphoenolpyruvate carboxykinase (PEPCK) enzyme activity and its mRNA levels in diabetic rats [19, 24]. PEPCK is the gluconeogenesis rate-controlling enzyme [25]. In most models of diabetes, PEPCK gene expression is elevated in the liver [26]. Studies have demonstrated that the enhanced activity of PEPCK contributes to increased blood sugar levels and diabetes exacerbation [27, 28]. Hyperglycemia is a common characteristic of diabetic conditions. It can result in the production of reactive oxygen species (ROS), which activates the stress-activated signaling pathways (including p38 mitogen-activated protein (MAP) kinase) and leads to insulin resistance [29]. In addition, oxidative stress has been found to upregulate hepatic PEPCK expression through an insulin-independent mechanism [30]. Gas chromatography/mass spectrometry (GC/MS) analysis of *SK* essential oil has demonstrated that its major components are phenolic compounds such as carvacrol and thymol in previous studies [31–33]. These compounds are known for their antioxidant properties [34]. Glucose-lowering, antioxidant, anti-inflammatory, and beneficial effects of carvacrol on diabetes have previously been reported in the animal model [35–38]. Several studies showed that thymol can decrease blood glucose levels in diabetic subjects [39, 40], and its antioxidant properties have been demonstrated [41]. The antioxidant defense system is compromised in type 2 diabetes, and the body’s incapacity to scavenge free radicals may contribute to tissue damage in the disease [42]. So, due to its antioxidant properties, SK can reduce the risk of diabetes complications.

Carvacrol can activate the expression of the transient receptor potential channel A1 (TRPA1) channel that stimulates glucagon-like peptide-1 (GLP-1) secretion [43]. GLP-1 lowers blood glucose levels by increasing glucose-dependent insulin secretion, reducing gastric emptying and postprandial glucagon levels, and increasing satiety [44]. Furthermore, it has been reported that carvacrol can increase the activity of hexokinase (HK) and 6-phosphofructokinase (PFK) enzymes [36]. So, it is hypothesized that carvacrol helps improve glucose metabolism by strengthening anaerobic glycolysis.

As mentioned above, in this study, *SK* showed lipid-lowering effects on diabetes patients. These beneficial effects may be related to its major constituents, carvacrol and thymol. It is reported that these compounds can enhance the activity of microsomal geranyl pyrophosphate pyrophosphatase enzyme twofold [45]. As well as they can lower cholesterol levels through competitive inhibition of 3-hydroxy-3-methylglutaryl coenzyme A (HMG-CoA) reductase [46], the rate-controlling enzyme of the mevalonate pathway. This enzyme catalyzes the conversion of HMG-CoA to mevalonic acid, an essential step in cholesterol biosynthesis [47]. It is reported that some polyphenols can inhibit the Niemann–Pick C1-like 1 (NPC1L1) protein and ultimately lower blood cholesterol levels [48]. It is a cell membrane protein located in the apical membrane of enterocytes and acts as a sterol transporter, promoting intestinal cholesterol absorption and balancing hepatobiliary cholesterol excretion [49]. Recent findings have shown that NPC1L1 deficiency or NPC1L1 inhibition reduces blood cholesterol and prevents hepatic steatosis and diet-induced obesity [50].

Compared to similar studies, the strengths of this study were a larger sample size, a higher dose of the *SK* supplement, and a longer duration of intervention, as well as recording dietary intakes using a 24-hour food recall questionnaire. In addition, the effects of the baseline variables (covariates) are adjusted in this study.

This study had the issue of generalizability as its most significant limitation. Compared with the general population of patients with type 2 diabetes, the study population appears to have less advanced disease due to the exclusion of people with diabetes taking insulin and patients with diagnosed micro- and macro-vascular diabetes complications.

## Conclusion

The findings of this study indicated that *SK* supplementation (500 mg per day) can improve glycemic indices (FBS, HbA1C, and Fasting Insulin) and lipid profiles (total cholesterol, LDL-c, and HDL-c) of type 2 diabetes patients. More studies and clinical trials with larger sample sizes and longer intervention duration are needed to clarify the exact mechanisms of this herbal supplement’s hypoglycemic and hypolipidemic effects.

## Data Availability

All data generated or analysed during this study are included in this published article.

## Abbreviations

SK: *Satureja Khuzestanica*
T2DM: Type 2 diabetes mellitus
HbA1c: Glycated hemoglobin
HDL: High-density lipo-protein
LDL: low-density lipo-protein
TG: Triglyceride
FBS: Fasting blood glucose
BMI: Body mass index
ALT: Alanine amino transferase
AST: Aspartate amino transferase
ELISA: Enzyme-linked immunosorbent assay
ANCOVA: Analysis of covariance
